# Awareness and Support of Release of Genetically Modified “Sterile” Mosquitoes, Key West, Florida, USA

**DOI:** 10.3201/eid2102.141035

**Published:** 2015-02

**Authors:** Kacey C. Ernst, Steven Haenchen, Katherine Dickinson, Michael S. Doyle, Kathleen Walker, Andrew J. Monaghan, Mary H. Hayden

**Affiliations:** University of Arizona, Tucson, Arizona, USA (K.C. Ernst, S. Haenchen, K. Walker);; National Center of Atmospheric Research, Boulder, Colorado, USA (K. Dickinson, M.H. Hayden, A.J. Monaghan);; Florida Keys Mosquito Control District, Key West, Florida, USA (M.S. Doyle)

**Keywords:** dengue, *Aedes aegypti*, OX513A, mosquitoes, dengue, viruses, genetically modified, sterile, awareness, support, Key West, Florida

## Abstract

After a dengue outbreak in Key West, Florida, during 2009–2010, authorities, considered conducting the first US release of male *Aedes aegypti* mosquitoes genetically modified to prevent reproduction. Despite outreach and media attention, only half of the community was aware of the proposal; half of those were supportive. Novel public health strategies require community engagement.

Two rapidly emerging viruses, chikungunya and dengue, are spread by *Aedes aegypti* mosquitoes (*1*)*.* Vector population control strategies have had variable success, and control by using genetically modified (GM) mosquitoes is under consideration (*2*)*.* In trials, 1 GM variant, the OX513A *Ae. aegypti*, has survived under field conditions and reduced wild-type populations (*3*,*4*). However, there were concerns among public health officials, ecologists, and entomologists that the measures used to engage and inform local communities were too limited (*5*,*6*). Community support has been linked to the success (*7*) and failure (*8*) of vector and pest control campaigns.

## The Study

During 2009–2010, an outbreak of dengue fever occurred in Key West, Florida (*9*). Shortly thereafter, the Florida Keys Mosquito Control District proposed the first release of a GM mosquito, OX513A *Ae. aegypti,* in the United States. The proposal was met with controversy.

On publication of this article, the release was undergoing inspection by the US Food and Drug Administration and had not occurred. 

We conducted a survey in June 2012 to examine awareness and support of the release after 80 media and outreach activities had been conducted in Key West and Stock Island, Florida. We randomly selected 400 residences from the Monroe County Property Appraisers Office database and administered a cross-sectional knowledge, attitudes, and practices survey about mosquito control and dengue virus. 

We collected information on demographics, perception of dengue risk, mosquito knowledge and prevention activities, and health care–seeking behavior, among other topics. Support was determined on a scale of 1 (strongly oppose) to 5 (strongly support). We requested reasons for participants’ level of support; themes raised by ≥9 respondents were coded into study categories by 2 investigators (K.C.E. and M.H.H.).

In this study, the use of GM male mosquitoes results in death of offspring in the larval or pupal stage of gestation; because of this outcome, outreach activities in the area preceding the survey referred to the mosquitoes as “sterile.” The survey we used included “sterile” because this term had been used in community awareness activities and should have been familiar to those who had heard of the proposed release, and we added “genetically modified” as a descriptor of the mosquitoes.

We divided participant groups into participants into those who had or had not heard of the release plans. We used logistic regression to assess associations between hearing of the release and possible explanatory factors. Missing values for household income were imputed. Distribution of levels of support of the release among those who had heard of the plan was stratified and tested for differences by demographic factors and participation in dengue and mosquito awareness and prevention activities. We used the Mantel-Haenszel test for trend for ordinal variables (e.g., education, income) and the χ^2^ test of heterogeneity for categorical variables. We used ANOVA, a nested analysis of variance approach, for continuous variables.

Of the 400 participants (Table 1), 75 (18.8%) were from the originally selected households. Of the 386 participants who responded to the question of whether they had heard of the proposed release before the survey, 195 (51.1%) answered “yes.” Prior awareness was more common in white non-Hispanics, residents with income levels >$50,000 per year, older adults, those who resided on Key West Island, and residents with knowledge of the local Action to Break the Cycle of Dengue public health campaign (Table 1). Among the 195 who were aware of the release, the distribution of support was: 9.7% strongly opposed, 8.2% opposed, 25.1% neutral, 22.1% supportive, and 34.9% strongly supportive. Men, less educated persons, and those willing to pay $100 or more for mosquito control were more likely to be strongly supportive (Table 2). The most common reasons for opposing the release were disturbance of nature and that it was an unproven technology. Most supporters of the release expressed a desire to do anything to get rid of mosquitoes or preferred the method to chemicals and spraying (Figure). On the basis of effectiveness, safety, and/or lack of unintended consequences, 22 of the 195 indicated that their support was conditional. 

**Table 1:** Comparison of 400 surveyed local residents who had heard of release of genetically modified “sterile” male *Aedes aegypti* OX513A mosquitoes to those who had not, Key West, Florida, USA*

Response	Sample distribution, no. (%)†	Heard, %‡	Not heard, %	Imputed unadjusted OR (95% CI), p value	Average adjusted OR (95% CI), multiply imputed data
Age, y					
18–35	98 (25.1)	19	81.1	1 (Referent)	1 (Referent)
36–50	77 (19.7)	55.3	44.7	5.16 (2.61–10.2), <0.001	3.75 (1.75–8.03), <0.001
51–65	121 (31.0)	71.8	28.2	10.5 (5.48–20.1), <0.001	8.17 (3.95–16.9), <0.001
>66	94 (24.1)	56.8	43.2	5.51 (2.84–10.7), <0.001	6.80 (3.14–14.7), <0.001
Sex					
M	214 (53.9)	56	44	1 (Referent)	1 (Referent)
F	183 (46.1)	45.3	54.8	0.65 (0.43–0.97), 0.03	0.58 (0.35–0.95), 0.03
Region of Key West					
Old Town	153 (38.4)	51	49	1 (Referent)	1 (Referent)
Midtown	61 (15.3)	55.2	44.8	1.17 (0.64–2.16), 0.60	1.33 (0.62–2.85), 0.46
New Town	126 (31.6)	57.4	42.6	1.29 (0.80–2.09), 0.29	1.53 (0.82–2.83), 0.18
Stock Island	59 (14.8)	33.9	66.1	0.49 (0.26–0.93), 0.03	0.65 (0.29–1.44), 0.29
Race/ethnicity					
White non-Hispanic	247 (66.9)	63	37	1 (Referent)	1 (Referent)
White Hispanic	46 (12.5)	36.4	63.6	0.33 (0.17–0.64), 0.001	0.47 (0.21–1.04), 0.06
Black	38 (10.3)	30.3	69.7	0.24 (0.11–0.53), <0.001	0.36 (0.15–0.90), 0.03
Other	38 (10.3)	27	73	0.21 (0.10–0.42), <0.001	0.25 (0.11–0.56), <0.001
Household income					
<$35,000	54 (13.5)	44	46	0.29 (0.14–0.60), <0.001	0.75 (0.31–1.82), 0.53
$35,000-$49,999	31 (7.8)	41.9	48.1	0.37 (0.15–0.90), 0.03	0.83 (0.30–2.30), 0.72
$50,000-$74,999	52 (13.0)	64.7	35.3	0.63 (0.30–1.32), 0.22	0.92 (0.41–2.08), 0.85
$75,000-$99,999	37 (9.3)	54.1	46	0.50 (0.22–1.15), 0.10	0.77 (0.31–1.92), 0.58
>$100,000	72 (18.0)	70.8	29.2	1 (Referent)	1 (Referent)
Education level					
High school or lower	123 (31.6)	34.8	65.2	1 (Referent)	1 (Referent)
Some college	77 (19.8)	45.3	54.7	1.54 (0.85–2.79), 0.15	1.71 (0.83–3.54), 0.15
Associate’s degree	19 (4.9)	68.4	31.6	3.83 (1.35–10.8), 0.01	5.73 (1.61–20.3), 0.007
Bachelor’s degree	107 (27.5)	55.7	44.3	2.38 (1.38–4.08), 0.002	1.93 (0.97–3.81), 0.059
Graduate or professional degree	63 (16.2)	77.8	22.2	6.63 (3.27–13.4), <0.001	3.37 (1.42–8.02), 0.006
Aware of ABCD§					
No	48 (19.0)	59.6	40.4	1 (Referent)	1 (Referent)
Yes	252 (81.0)	79.2	20.8	2.56 (1.27– 5.14), 0.008	2.32 (1.04–5.17), 0.04

*All variables listed are included in the adjusted model. †Demographic totals may not add up to 400 because some participants refused to report demographic information. ‡Percentages reflect within category percentages. §ABCD, Florida Keys-based Action to Break the Cycle of Dengue public health campaign.

**Table 2:** Percentage of responses to demographic, dengue and mosquito-related factors according to level of support for a release of genetically modified “sterile” mosquitoes in Key West, Florida, USA, among the 195 participants who had heard of the release*

Response	Strongly opposed	Somewhat opposed	Neutral	Somewhat supportive	Strongly supportive	p value
Overall level of support, no. (%)	19 (9.7)	16 (8.2)	49 (25.1)	43 (22.1)	68 (34.9)	NA
Mosquitoes noticed outside (%, many or very many)	26.3	12.5	14.6	11.9	22.1	0.87†
How many days did you spend outside last week, % >3 d	79.0	68.8	83.7	79.1	75.0	0.75†
Limit outdoor activity because of mosquitoes, % often or always	10.5	6.3	10.2	4.7	8.8	0.80†
Able to report dengue as a mosquito-carried disease, % yes	84.2	87.5	75.5	79.1	80.9	0.78†
How serious is dengue in Key West, % very or extremely serious	31.6	43.8	38.6	40.0	31.3	0.63†
How likely is it that you or a family member will get dengue in Key West, % somewhat or very likely	10.5	18.8	10.6	12.8	10.8	0.78†
Aware of ABCD, % yes	15.8	18.8	26.1	29.0	14.3	0.68†
Willing to pay $100 or more for effective mosquito control, %, yes	28.6	50.0	58.7	73.5	73.3	<0.001†
Current mosquito control is very or extremely effective, % yes	66.7	75.0	75.5	69.8	72.1	0.97†
Mean age, y	57.8	52.6	54.7	56.2	57.7	0.67‡
Distribution of support by category						
Sex						<0.001§
M	10.5	6.1	18.4	17.5	47.4	ND
F	8.6	11.1	34.6	28.4	17.3	ND
Key West region						0.29§
Old Town	13.3	8.0	25.3	20.0	33.3	ND
Midtown	3.1	9.4	34.4	25.0	28.1	ND
New Town	7.3	7.3	26.1	17.4	42.0	ND
Stock Island	15.8	10.5	5.3	42.1	26.3	ND
Race						0.21§
White non-Hispanic	9.2	8.5	24.2	20.9	37.3	ND
White Hispanic	6.3	0.0	18.8	37.5	37.5	ND
Black	0.0	10.0	50.0	20.0	20.0	ND
Other race	30.0	20.0	10.0	20.0	20.0	ND
Household income						0.16†
<$35,000	13.6	13.6	18.2	27.3	27.3	ND
$35,000-$49,999	7.7	15.4	7.7	46.2	23.1	ND
$50,000-$74,999	12.5	3.1	21.9	18.8	43.8	ND
$75,000-$99,999	10.0	10.0	30.0	25.0	25.0	ND
>$100,000	3.9	7.8	27.5	13.7	47.1	ND
Highest level of education						0.09†
Lower than high school	0.0	0.0	25.0	25.0	50.0	ND
High school graduate	0.0	5.6	19.4	25.0	50.0	ND
Some college	15.6	12.5	25.0	25.0	21.9	ND
Associate degree	7.7	0.0	30.8	23.1	38.5	ND
Bachelor’s degree	11.9	10.2	28.8	18.6	30.5	ND
Graduate or professional degree	12.2	6.1	24.5	20.4	36.7	ND

*NA, not applicable; ND, calculation not done; ABCD, Florida Keys-based Action to Break the Cycle of Dengue public health campaign. †p value for trend calculated by using Mantel-Haenszel test. ‡p value calculated by using nested analysis of variance. §p value calculated by using χ^2^ test for heterogeneity.

**Figure:**
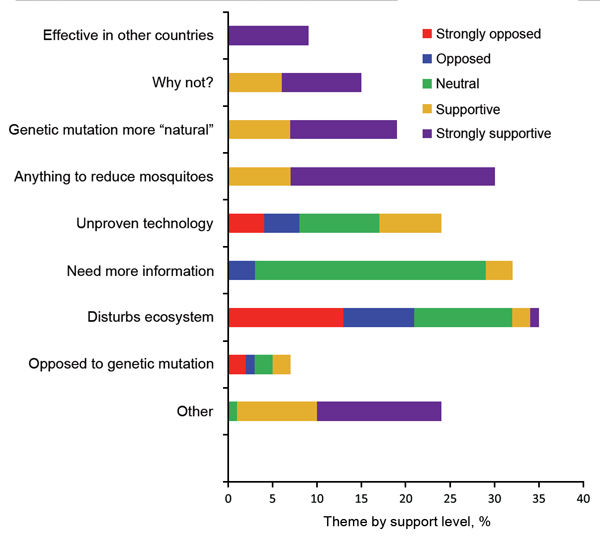
Proportion of respondents reporting different themes for their level of support of plans to release genetically modified, referred to as “sterile,” male mosquitoes on Key West, Florida, USA.

## Conclusions

For community acceptance of the release of GM mosquitoes, several issues must be addressed. Release of GM mosquitoes into the community should be transparent; therefore, the Florida Keys Mosquito Control District has begun to disseminate information through public events, articles, and presentations. Identification of solutions to reduce risk for vector-borne disease should involve stakeholders from the public, and community leaders in public health, vector control, and municipal administrators. Open communication with community members and stakeholders through a health advisory board was instrumental in quelling a 1989 invasion of Mediterranean fruit flies in California that had become a crisis event (*10*). In Key West and Stock Island, public awareness and communication campaigns had limited success. Awareness of the release varied across sections of the city and by demographic group. At the time of the survey, the release was planned for Key West; in Stock Island, awareness was much lower. Adjacent areas should be included in communications because residents and *Ae. aegypti* are mobile. (*11*)*.* Knowledge of current events has been associated with gender, education level, race and ethnicity, and age (*12*). Outreach should target groups with a tendency towards lower awareness of public health measures.

Support was more commonly reported than opposition among those aware of the release; a large portion was neutral. Most neutral respondents reported they did not know enough to make a decision, and many supporters wanted more information or had concerns. To progress from awareness to knowledge, to understanding, and then to decision-making would require considerable effort and improvement in overall scientific literacy (*13*). The scientific community is divided about the amount of information that should be provided to community members on highly technical vector control strategies such as the release of the OX513A mosquito (*14*). Benchmarks for acceptable engagement and support should be set by public health organizations before GM vector releases are planned, which will require input from scientists, stakeholders, and the community.

Strongly opposed participants most commonly reported unintended consequences or disturbing natural ecosystems as their reason for opposition. Conversely, some supporters considered the mosquito release a more natural way of controlling mosquito populations than insecticides. This was substantiated in a follow-up study (*15*).

This study has several limitations. Participants may not have fully represented the community because of seasonal housing closures and inaccessibility of some gated communities. A systematic replacement strategy was used to minimize bias. To obtain information on support, we provided a short statement about the release, modeled after earlier community outreach efforts and that used the term “sterile mosquito” instead of “genetically modified mosquito.” We excluded responses of participants without prior awareness from our analysis because our informational statement was cursory. Follow-up studies in Key West that provided more extensive information yielded the same 9% strong opposition rate (*15*).

Introduction of GM mosquitoes has the potential to reduce mosquito-borne disease; however, little data exist on the type and extent of outreach required or community support needed to reduce opposition. As of December 2014, a short-term release of Oxitec OX513A mosquitoes is proposed on Key Haven, a peninsula adjacent to Key West. This is part of an application by Oxitec: Regulatory Clearance for Investigational Use of a New Animal Drug. This release is proposed before broader implementation in Key West or elsewhere in the Florida Keys (M.S. Doyle, unpub. data). If approved, this release could serve as a model of best practices for establishing community relations and engagement before implementing vector control strategies.
